# Metagenomic Identification of a Novel Zoonotic Gemykibivirus in a Diarrheic Pig in China

**DOI:** 10.1155/tbed/7560012

**Published:** 2025-07-24

**Authors:** Wenqiang Wang, Lin Yuan, Miaojie Zhang, Qilin Zhao, Wenqiang Pang, Dehu Sun, Xiaoye Jia, Feifei Tan, Tingting Niu, Kegong Tian, Xiangdong Li

**Affiliations:** ^1^Jiangsu Co-innovation Center for Prevention and Control of Important Animal Infectious Diseases and Zoonoses, College of Veterinary Medicine, Yangzhou University, Yangzhou, Jiangsu Province, China; ^2^Beijing Sino-science Gene Technology Co. Ltd., Beijing, China; ^3^National Research Center for Veterinary Medicine, Luoyang, Henan Province, China; ^4^Pulike Biological Engineering Inc., Luoyang, Henan Province, China

**Keywords:** gemykibivirus, metagenomic, porcine, zoonoses

## Abstract

Gemykibiviruses are circular, replication-associated protein (REP) encoding single-stranded DNA (ssDNA) viruses classified within the genus *Gemykibivirus* of the family Genomoviridae. In recent years, several gemykibiviruses have been detected in humans presenting with clinical symptoms, such as encephalitis, respiratory illness, sepsis, pericarditis, diarrhea, and multiple sclerosis. However, the presence of gemykibiviruses in other animal hosts, as well as the evolution of the *Gemykibivirus* genus, remains poorly understood. In this study, we identified a novel gemykibivirus from a diarrheic pig in China using metagenomic sequencing, which we designated as pGkV. The pGkV genome comprises 2200 nucleotides and encodes two key proteins as follows: the capsid-associated protein (CAP) and the REP. Phylogenetic analyses revealed that pGkV clusters within the zoonotic gemykibivirus (ZooGkV) clade, which includes nearly all gemykibivirus species recently identified in humans. Mutation and haplotype analyses revealed that pGkV is closely related to an avian gemykibivirus, while its CAP and REP proteins are identical to those of a human gemykibivirus, underscoring its potential zoonotic capability. Furthermore, recombination signals were detected among Zoo-GkVs, suggesting that recombination may contribute to the emergence of novel gemykibivirus strains. The identification of pGkV provides new insights into the evolution and cross-species transmission of gemykibiviruses.

## 1. Introduction

Metagenomic next-generation sequencing (mNGS) has substantially enhanced the efficiency of novel pathogen discovery due to its nontargeted, high-throughput nucleic acid detection capabilities, particularly in cases of complex infections and outbreaks caused by unknown pathogens [1]. This was exemplified by its pivotal role in the rapid identification of SARS-CoV-2 during the COVID-19 pandemic, with the virus being characterized within 40 h [2]. By enabling unbiased detection of both known and previously unrecognized viruses through comprehensive nucleic acid sequencing, mNGS has greatly expanded our understanding of viral diversity [3, 4].

The family Genomoviridae, established by the International Committee on Taxonomy of Viruses (ICTV) in 2016, constitutes a distinct lineage of circular Rep-encoding single-stranded DNA (CRESS-DNA) viruses [5]. These viruses are characterized by small, monopartite, nonenveloped genomes encapsidated within icosahedral particles measuring approximately 20–22 nm in diameter. Genomic analyses have revealed that members of this family possess compact circular ssDNA genomes ranging from 1.8 to 2.4 kilobases (kb) in length [5]. Currently, Genomoviridae comprises 10 genera, including *Gemykibivirus, Gemycircularvirus, Gemyduguivirus, Gemygorvirus, Gemyvongvirus, Gemykolovirus, Gemykrogvirus, Gemytripvirus, Gemykroznavirus*, and *Gemytondvirus* [5, 6]. These viruses typically encode two major open reading frames (ORFs): one for a replication-associated protein (REP), which contains conserved endonuclease and helicase domains, and another for a capsid protein (CAP), often expressed via spliced transcripts. Phylogenetically, the Genomoviridae family forms a sister clade to the Geminiviridae, with species demarcation defined by less than 78% whole-genome sequence identity [6].

Viruses of the *Gemykibivirus* genus have been predominantly detected in various human clinical cases, across a wide range of sample types, including those associated with encephalitis, respiratory diseases, sepsis, pericarditis, diarrhea, and multiple sclerosis [7–9]. However, a direct causal relationship between gemykibivirus infection and these clinical manifestations has not been firmly established. The occurrence of gemykibiviruses in nonhuman hosts and the mechanisms underlying their cross-species transmission remain poorly understood. In this study, we identified a novel gemykibivirus from a diarrheic pig in China using mNGS technology. We further conducted a comprehensive evolutionary analysis of viruses within the *Gemykibivirus* genus. Our findings provide new evidence supporting the potential zoonotic capacity of this newly identified porcine gemykibivirus (pGKV), highlighting the importance of continued surveillance, and characterization of gemykibiviruses in both human and animal hosts.

## 2. Materials and Methods

### 2.1. Sample Collection, DNA Extraction, and Sequencing

Tissue sample was homogenized in sterile PBS (90 Hz, 90s) and centrifuged at 3000 rpm for 5 min. A 0.2 mL aliquot of the supernatant was collected, with porcine kidney cells (PK-15) serving as the negative control. Total DNA and RNA were coextracted using the MagMAX CORE Nucleic Acid Extraction Kit via magnetic bead-based purification. Nucleic acid concentrations were quantified using the Equalbit 1×dsDNA HS Assay Kit and Equalbit RNA HS Assay Kit. Library construction was performed according to the protocol provided by the manufacturer of the MGIEasy Fast Enzymatic Fragmentation Library Prep Kit v2.0. DNA nanoballs (DNBs) were prepared using the MGISEQ-2000RS High-Throughput Rapid Sequencing Kit. Sequencing was conducted on the MGISEQ-2000 platform with a single-end 100 bp (SE100) read length.

### 2.2. Data Processing

Sequencing reads were first adaptor- and quality-trimmed using the Fastp (v 0.23.2) program with default parameters [10]. After quality control, the clean data were further aligned to the pig genome (GCF_000003025.6) to remove the host genome using bowtie2 (v2.3.5.1) program [11]. The remaining reads were assembled de novo using SPAdes (v3.15.5) program with default parameter settings [12]. Assembled contigs were compared against the NCBI core nucleotide database (core nt) using online BLASTn. A CRESSV-like virus with genome length 2200 nucleotides were identified.

### 2.3. Phylogenetic Analyses

In order to further elucidate the new identified CRESSV belongs to which CRESSV family, we integrated the REP protein of the new virus with the REP sequences from various families, including Circoviridae, Smacoviridae, Nanoviridae, Geminiviridae, Genomoviridae, Redondoviridae, and CRESSV1~6. The virus sequences were aligned using the L-INS-i algorithm implemented in the program MAFFT (v7.518) [13]. Maximum-likelihood phylogenies were inferred from the amino acid alignments using IQ-TREE (v 2.2.0) under the best-fit substitution model, automatically selected via the -m MFP option. Branch support was assessed with 1000 replicates of the SH-like approximate likelihood ratio test (SH-aLRT) [14]. Upon confirming the classification of the novel virus within the Genomoviridae family, we proceeded to further characterize and validate its genus-level taxonomic assignment. We selected represent sequences from all 10 genus of *Genomoviridae*. MAFFT (v7.518) and IQ-TREE (v 2.2.0) were used to align REP protein sequences and constructed phylogenetic relationships, respectively. In order to investigate the evolution of viruses in genus *Gemykibivirus* and identify the phylogenetic position of the novel virus among the genus, we download all *Gemykibivirus* sequences from genbank in NCBI and further validated the taxonomic assignment of these Gemykibiviruses by phylogenetic analyses. The REP protein sequences of all characterized Gemykibiviruses, including the novel isolate, were aligned using MAFFT and subsequently employed to construct a maximum-likelihood phylogenetic tree with IQ-TREE.

### 2.4. Mutation and Haplotype Analyses

To evaluate genomic similarity among zoonotic gemykibiviruses (ZooGkVs), we performed pairwise alignments between the novel virus, which served as the reference genome, and each of the other known Zoo-GkVs using MAFFT [13]. Genetic variations, including single-nucleotide polymorphisms (SNPs), insertions, and deletions, were identified and compiled into a variant call format (VCF) file using a custom Python script. Subsequently, a genome-wide mutation map was visualized using an R-based package. Haplotype network analyses were also employed in this study. The genome sequences of all Zoo-GkVs were aligned using MAFFT, and the resulting multiple sequence alignment file was subsequently analyzed in DNAsp to generate a haplotype data file [15]. Haplotype network analysis was then performed in POPART utilizing the median-joining algorithm [16].

### 2.5. Recombination Analyses

To elucidate the role of recombination in the Zoo-GkVs, we conducted comprehensive recombination analyses across all Zoo-GkVs. The multiple sequence alignments originally constructed for haplotype characterization were repurposed for recombination detection analyses in the current investigation. To detect potential recombination events, we employed SplitsTree software to construct phylogenetic networks for visualizing recombination signals [17]. Additionally, we performed Pairwise Homoplasy Index (PHI) tests to statistically assess recombination events across the genome, with a significance threshold of *p*  < 0.05 for identifying evolutionarily significant recombination patterns.

## 3. Results

### 3.1. Discovery and Genome Annotation of a New Virus in Swine

**Figure 1:**
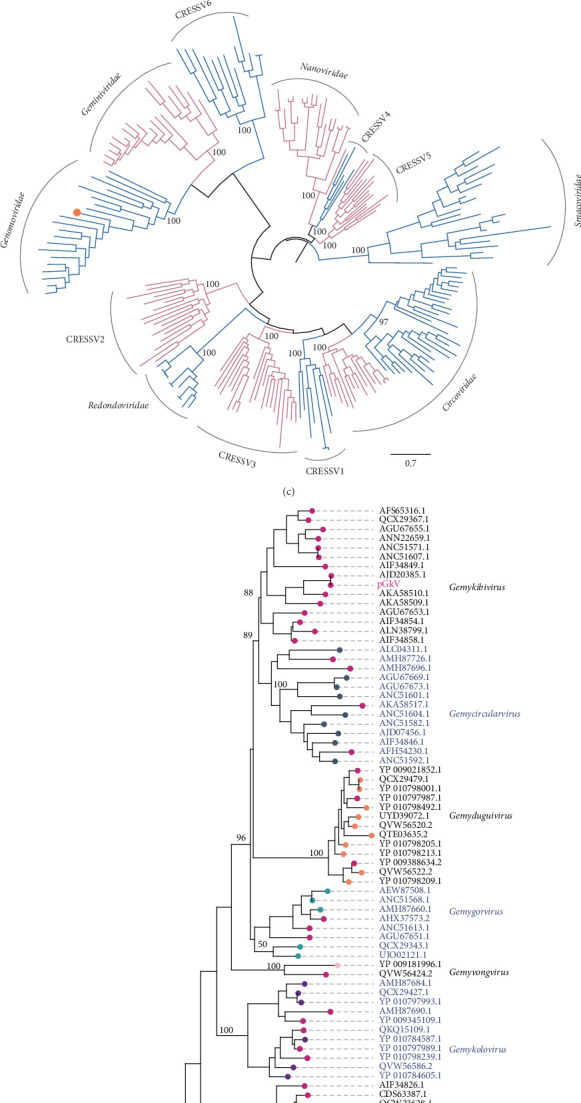
Genome organization and phylogenetic classification of pGkV. (A) Schematic diagram of the gemykibivirus genome organization. (B) The origin of replication (ori) of pGkV. (C) Maximum-likelihood phylogeny of CRESS DNA viruses based on REP protein sequences, where the pGkV sequence is marked with an orange dot. (D) Phylogenetic reconstruction of Genomoviridae members using REP sequences, with pGkV indicated by a pink marker.

mNGS has become an increasingly important tool in pathogen identification by enabling the comprehensive analysis of microbial and host genetic material within a given sample. In this study, mNGS was employed to identify pathogens in a diarrheic pig, leading to the complete sequencing of a new circular single-stranded DNA virus genome. The viral genome is 2200 nucleotides in length and features a nonanucleotide motif [TAAAATTTA], rich in adenine (A) and thymine (T), at the apex of the stem-loop structure (Figure 1). Gene annotation revealed that the circular virus harbors two genes arranged in opposite orientations; one encoding the CAP protein on the positive strand and the other encoding the REP protein on the negative strand, with an intron located in the rep gene (Figure 1A). The genomic features are consistent with those of CRESS-DNA viruses, supporting the classification of this virus within the CRESS-DNA group.

### 3.2. Phylogenetic Analyses of Gemykibiviruses

Given that porcine circoviruses, also members of the CRESS DNA virus group, have caused substantial economic losses in the pig industry, determining the phylogenetic position of the newly identified CRESS DNA virus is of particular relevance. To this end, we selected 216 REP protein sequences from 12 major CRESS DNA virus families, including the families Circoviridae, Redondoviridae, Genomoviridae, Geminiviridae, Nanoviridae, Smacoviridae, and CRESSV1~6, and integrated them with the Rep sequence of the CRESS DNA virus identified in this study to construct a phylogenetic tree. Phylogenetic analysis revealed that the newly identified CRESS DNA virus and the members of the Genomoviridae family form a stable monophyletic group, strongly supported by a bootstrap value of 100 (Figure 1C). Upon confirming the virus's affiliation with Genomoviridae family, we selected 84 representative REP protein sequences from this family, covering all genera within the family, and combined them with the new virus identified in this study to construct the phylogenetic tree. Results demonstrated that the new virus clusters within the *Gemykibivirus* genus with a bootstrap value of 88 (Figure 1D). Based on the phylogenetic findings, we have designated the newly identified CRESS DNA virus as pGkV.

**Figure 2:**
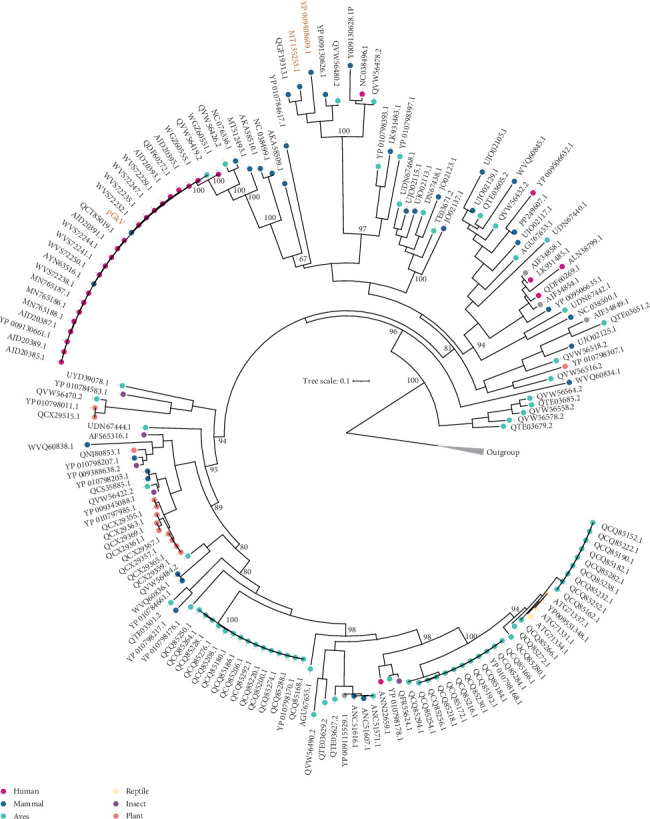
Phylogenetic analyses of the Gemykibivirus genus. Phylogenetic reconstruction of gemykibivirus members based on REP sequences, with porcine gemykibiviruses marked in brown. The host range of gemykibiviruses is distinguished by different-colored dots.

To advance our understanding of the diversity within the *Gemykibivirus* genus and to clarify the taxonomic position of pGkV, we constructed a phylogenetic tree based on REP protein sequences from all gemykibiviruses reported to date. The analysis revealed that the *Gemykibivirus* genus exhibits a broad host spectrum, encompassing humans, other mammals, birds, reptiles, insects, and plants (Figure 2). Gemykibiviruses from different host groups consistently formed distinct, stable monophyletic clusters within the phylogenetic tree. This widespread distribution of monophyletic groups underscores not only the significant diversity within the *Gemykibivirus* genus but also its strong potential for cross-host transmission (Figure 2). Notably, pGkV, identified in this study, is not closely related to the two previously reported pig gemykibiviruses. Instead, it clustered with a group of human gemykibiviruses with strong phylogenetic support (bootstrap value = 100), and exhibited short branch lengths within the clade, indicating a close genetic relationship among these gemykibiviruses (Figure 2). We designated this group as the “Zoo-GkV”. These findings suggest that pGkV likely represents a novel member of this emerging zoonotic lineage.

### 3.3. Evolutionary Insights and Cross-Host Affinities of pGkV

**Figure 3:**
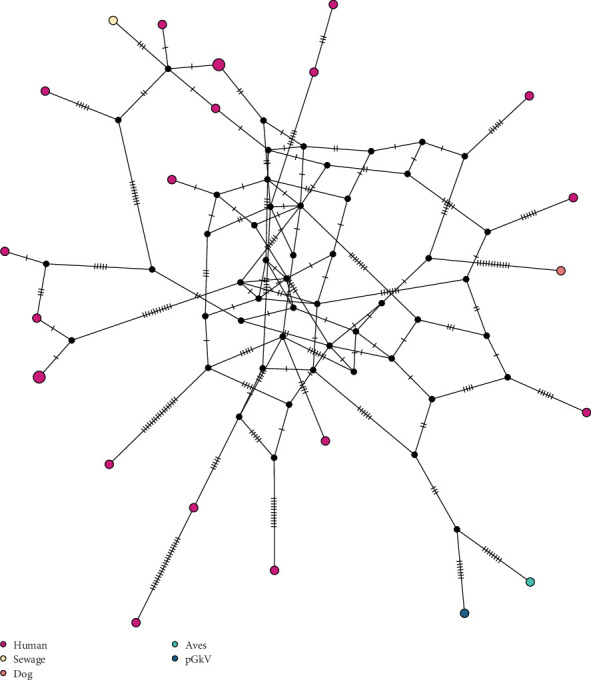
Haplotype network of zoonotic gemykibiviruses. Haplotype network analyses of zoonotic gemykibiviruses based on complete genome sequences. The host range or sample origin of gemykibiviruses is distinguished by different-colored dot.

**Figure 4:**
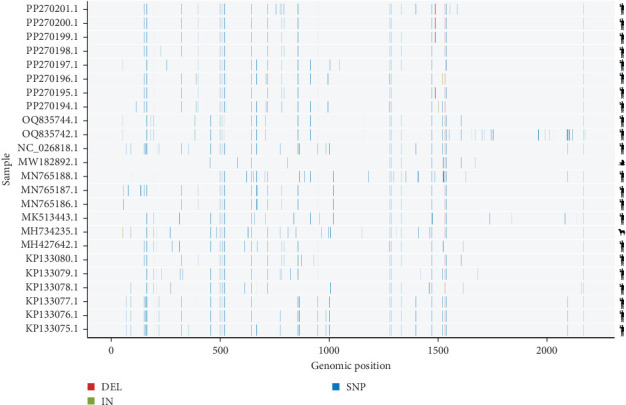
Mutation atlas of zoonotic gemykibiviruses. Genome-wide variation among zoonotic gemykibivirus members compared to pGkV. SNPs, insertions, and deletions are indicated by different color. Host species corresponding to each gemykibivirus are illustrated by graphical symbols on the right side.

**Figure 5:**
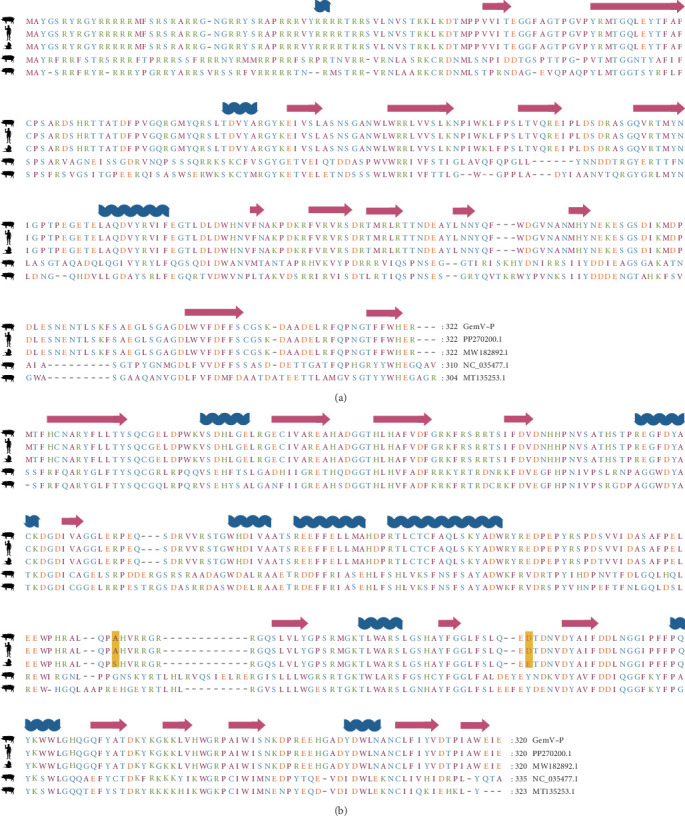
Protein alignments of gemykibiviruses. (A) Alignment of the CAP protein sequences of pGkV with human gemykibivirus (PP270195.1), avian gemykibivirus (MW182892.1), and two previously reported porcine gemykibiviruses (NC_035477.1 and MT135253.1). (B) Alignment of the REP protein sequences of pGkV with human gemykibivirus (PP270195.1), avian gemykibivirus (MW182892.1), and two previously reported porcine gemykibiviruses (NC_035477.1 and MT135253.1). Secondary structural elements, including α-helices and β-sheets, are illustrated with corresponding diagrams above the alignments.

Due to the high sequence similarity of REP proteins among Zoo-GkV members, the precise phylogenetic placement of pGkV within this clade could not be fully resolved. To further elucidate the evolutionary relationship between pGkV and other Zoo-GkV members, we constructed a haplotype network based on SNPs derived from whole-genome alignments. The analysis revealed that pGkV is most closely related to a bird-associated gemykibivirus (Figure 3). To gain a more detailed view of the genomic variation, we performed pairwise genome alignments using pGkV as the reference, providing a comprehensive overview of sequence divergence between pGkV and other Zoo-GkV strains. The mutation landscape revealed that the first half of the pGkV genome exhibits the highest similarity to a bird-derived gemykibivirus, differing by only five SNPs, while the latter half shows greater similarity to several human-associated gemykibiviruses (Figure 4). We then compared the CAP and REP proteins of pGkV with four representative gemykibiviruses and found that neither the CAP nor the REP protein of pGkV showed high similarity to the previously reported swine viruses, consistent with the earlier phylogenetic results (Figure 5). Although haplotype network displays that pGkV is most closely related to the bird-associated gemykibivirus, protein alignments reveal that the REP and CAP proteins are completely identical to the human gemykibivirus, further indicating the zoonotic ability of pGkV (Figure 5). The CAP protein of the bird-associated gemykibivirus is identical to that of pGkV, whereas its REP protein differs by two amino acid residues (Figure 5).

### 3.4. Recombination Contributes to the Emergence of New Gemykibiviruses

**Figure 6:**
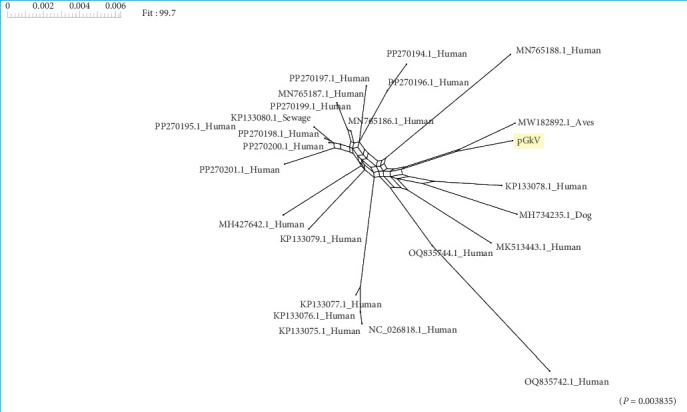
Recombination network of zoonotic gemykibiviruses. Genome sequences of zoonotic gemykibiviruses were analyzed for recombination events using SplitsTree software.

Given that the haplotype network indicates that pGkV is most closely related to the bird-associated gemykibivirus, while protein alignments reveal that proteins of pGkV are absolutely identical to the human gemykibivirus, we infer that the Zoo-GkVs may have experienced recombination. To validate the hypothesis, we performed recombination analyses across these Zoo-GkVs. Recombination analysis revealed a distinct network topology among Zoo-GkVs, indicating the occurrence of recombination events (Figure 6). This finding was further substantiated by the PHI test, which confirmed the statistical significance of these recombination events (*p*=0.0038).

## 4. Discussion

In this study, we employed mNGS to identify a gemykibivirus (pGkV) from a diarrheic pig in China. The complete genome sequence of pGkV has been deposited in the NCBI GenBank database under the Accession Number PV329706.1. The identified pGkV is not closely related to the two previously reported pGkVs, but instead forms a stable monophyletic group with some human gemykibiviruses with a bootstrap value of 100, suggesting that pGkV is a new gemykibivirus and not derived from the previously reported pGkVs (Figure 2). The pGkV related cluster was named Zoo-GkV, which also contains a dog associated gemykibivirus and a bird associated gemykibivirus. Zoo-GkV contains most of the human-associated gemykibiviruses reported to date, which were mainly identified in patients with some symptoms, including encephalitis, diarrhea, febrile, and acute respiratory distress syndrome [18–20]. Regrettably, although many gemykibiviruses were identified in patients with obvious symptoms, direct evidence supporting members of the genus *Gemykibivirus* as causative agents of disease is still lacking. In this study, the pGkV-positive sample also contained other viruses, including African swine fever virus, adenovirus, and coronavirus, indicating a coinfection scenario. Hence, whether the diarrhea observed in the pig was caused by pGkV remains unclear.

Although the haplotype network displayed that pGkV is most closely related to the bird associated gemykibivirus (MW182892.1), the two encoded proteins, REP and CAP, are completely identical to those of a human gemykibivirus (PP270195.1), indicating that pGkV may possess the ability to infect humans without requiring any genetic variation (Figure 5). This raises the possibility of cross-species transmission, although the exact routes and mechanisms remain to be determined. The identification of Zoo-GkV in patients from Nepal, China, France, and Brazil suggests that gemykibiviruses may be globally disseminated [18, 20–22]. Zoo-GkV was initially detected in 2017 in China, where it was identified in a throat swab specimen collected from a woman suffering from unexplained acute respiratory distress syndrome [20]. Together with the identification of pGkV in China, these data indicate that the Zoo-GkVs have been circulating in China for nearly a decade, and even exhibit different host tropisms. In addition, we detected pGkV-related reads in another clinical sample, although a complete genome could not be assembled, suggesting that pGkV may not be an isolated case. With the help of metagenomic NGS, an increasing number of gemykibiviruses are expected to be discovered in the future.

Genome and recombination analyses reveal recombination events in Zoo-GkV, which provide genetic material for the evolution of gemykibiviruses and may act as an important driving force for the emergence of new gemykibivirus strains. Given the fact that coinfection with multiple viruses is the prerequisite for recombination, the existence of recombination events in Zoo-GkV indicates occurrence of gemykibivirus coinfection. The phylogenetic tree of gemykibiviruses constructed in this study is the most detailed representation of the *Gemykibivirus* genus to date, and this genus comprises viruses that have been detected in various parts of the world, associated with various organisms, including mammals, birds, reptiles, insects, and plants. Notably, the host range of the Zoo-GkV lineage includes both mammals and aves. Recombination analyses were employed only across the Zoo-GkVs in this study, and more recombination events may exist in the broader *Gemykibivirus* genus.

One limitation of this study is that the pGkV was not successfully isolated, despite attempts using standard protocols for circovirus cultivation, thereby limiting our knowledge of the pathogenic role of pGkV. Not only has pGKV not been successfully isolated, but no members of the *Gemykibivirus* genus have been isolated and utilized in animal experimentation to date. Hence, there is an urgent need to isolate *gemykibivirus* and elucidate transmission routes, host interactions and pathogenesis. Although a clear lack of direct evidence supporting the genus *Gemykibivirus* as a potential candidate for disease causation exists, many *gemykibiviruses* were identified in patients and animals with diseases, indicating their potential pathogenicity [18–20, 23]. Nevertheless, this study identified a new gemykibivirus and assessed its potential zoonotic risk, providing novel insight into the evolution of *Gemykibivirus* genus.

## 5. Conclusion

In this study, we identified and characterized a novel gemykibivirus, designated pGkV, from a diarrheic pig in China. Genomic and phylogenetic analyses demonstrated that pGkV belongs to the Zoo-GkV clade and shares high similarity with both avian and human strains, particularly in its CAP and REP protein sequences, highlighting its potential zoonotic nature. Moreover, the detection of recombination signals among Zoo-GkVes points to recombination as a potential evolutionary mechanism driving the emergence of novel strains. Together, our results expand the known host range of gemykibiviruses and provide important insights into their evolution and cross-species transmission dynamics, underscoring the need for enhanced surveillance of these viruses in both human and animal populations.

## Data Availability

The data that support the findings of this study are available from the corresponding author upon reasonable request.

## References

[1] Chiu C. Y., Miller S. A. (2019). Clinical Metagenomics. *Nature Reviews Genetics*.

[2] Wu F., Zhao S., Yu B. (2020). A New Coronavirus Associated With Human Respiratory Disease in China. *Nature*.

[3] Chen Y. M., Hu S. J., Lin X. D. (2023). Host Traits Shape Virome Composition and Virus Transmission in Wild Small Mammals. *Cell*.

[4] Hou X., He Y., Fang P. (2024). Using Artificial Intelligence to Document the Hidden RNA Virosphere. *Cell*.

[5] Krupovic M., Ghabrial S. A., Jiang D., Varsani A. (2016). Genomoviridae: A New Family of Widespread Single-Stranded DNA Viruses. *Archives of Virology*.

[6] Varsani A., Krupovic M. (2017). Sequence-Based Taxonomic Framework for the Classification of Uncultured Single-Stranded DNA Viruses of the Family *Genomoviridae*. *Virus Evolution*.

[7] Halary S., Duraisamy R., Fancello L. (2016). Novel Single-Stranded DNA Circular Viruses in Pericardial Fluid of Patient With Recurrent Pericarditis. *Emerging Infectious Diseases*.

[8] Macera L., Spezia P. G., Medici C. (2019). Low Prevalence of Gemycircularvirus DNA in Immunocompetent and Immunocompromised Subjects. *The New Microbiologica*.

[9] Bezerra R. S., Bitencourt H. T., Covas D. T., Kashima S., Slavov S. N. (2020). Metagenomic Identification of Human Gemykibivirus-2 (HuGkV-2) in Parenterally Infected Blood Donors From the Brazilian Amazon. *International Journal of Infectious Diseases*.

[10] Chen S., Zhou Y., Chen Y. (2018). Gu J: Fastp: An Ultra-Fast All-in-One FASTQ Preprocessor. *Bioinformatics*.

[11] Langmead B., Salzberg S. L. (2012). Fast Gapped-Read Alignment With Bowtie 2. *Nature Methods*.

[12] Bankevich A., Nurk S., Antipov D. (2012). SPAdes: A New Genome Assembly Algorithm and its Applications to Single-Cell Sequencing. *Journal of Computational Biology*.

[13] Katoh K., Standley D. M. (2013). MAFFT Multiple Sequence Alignment Software Version 7: Improvements in Performance and Usability. *Molecular Biology and Evolution*.

[14] Minh B. Q., Schmidt H. A., Chernomor O. (2020). IQ-TREE 2: New Models and Efficient Methods for Phylogenetic Inference in the Genomic Era. *Molecular Biology and Evolution*.

[15] Rozas J., Ferrer-Mata A., Sanchez-DelBarrio J. C. (2017). DnaSP 6: DNA Sequence Polymorphism Analysis of Large Data Sets. *Molecular Biology and Evolution*.

[16] Leigh J. W., Bryant D. (2015). POPART: Full-Feature Software for Haplotype Network Construction. *Methods in Ecology and Evolution*.

[17] Huson D. H., Bryant D. (2024). The SplitsTree App: Interactive Analysis and Visualization Using Phylogenetic Trees and Networks. *Nature Methods*.

[18] Tuladhar E. T., Shrestha S., Vernon S. (2024). Gemykibivirus Detection in Acute Encephalitis Patients From Nepal. *mSphere*.

[19] Phan T. G., Mori D., Deng X. (2015). Small Circular Single Stranded DNA Viral Genomes in Unexplained Cases of Human Encephalitis, Diarrhea, and in Untreated Sewage. *Virology*.

[20] Wang J., Li Y., He X. (2019). Gemykibivirus Genome in Lower Respiratory Tract of Elderly Woman With Unexplained Acute Respiratory Distress Syndrome. *Clinical Infectious Diseases*.

[21] Silverio B. S., Sanz Duro R. L., de Sousa L. L. F. (2025). Detection and Phylogenetic Analysis of Emerging Human-Associated Gemykibivirus-2 in *Molossus molossus* Bat From Brazil. *Journal of Medical Virology*.

[22] Uch R., Fournier P. E., Robert C. (2015). Divergent Gemycircularvirus in HIV-Positive Blood, France. *Emerging Infectious Diseases*.

[23] Zhou C., Zhang S., Gong Q., Hao A. (2015). A Novel Gemycircularvirus in an Unexplained Case of Child Encephalitis. *Virology Journal*.

